# Adolescent Engagement in Dangerous Behaviors Is Associated with Increased White Matter Maturity of Frontal Cortex

**DOI:** 10.1371/journal.pone.0006773

**Published:** 2009-08-26

**Authors:** Gregory S. Berns, Sara Moore, C. Monica Capra

**Affiliations:** 1 Department of Psychiatry & Behavioral Sciences, Emory University School of Medicine, Atlanta, Georgia, United States of America; 2 Economics Department, Emory University, Atlanta, Georgia, United States of America; 3 Center for Neuropolicy, Emory University, Atlanta, Georgia, United States of America; University of Granada, Spain

## Abstract

**Background:**

Myelination of white matter in the brain continues throughout adolescence and early adulthood. This cortical immaturity has been suggested as a potential cause of dangerous and impulsive behaviors in adolescence.

**Methodology/Principal Findings:**

We tested this hypothesis in a group of healthy adolescents, age 12–18 (N = 91), who underwent diffusion tensor imaging (DTI) to delineate cortical white matter tracts. As a measure of real-world risk taking, participants completed the Adolescent Risk Questionnaire (ARQ) which measures engagement in dangerous activities. After adjusting for age-related changes in both DTI and ARQ, engagement in dangerous behaviors was found to be positively correlated with fractional anisotropy and negatively correlated with transverse diffusivity in frontal white matter tracts, indicative of increased myelination and/or density of fibers (ages 14–18, N = 60).

**Conclusions/Significance:**

The direction of correlation suggests that rather than having immature cortices, adolescents who engage in dangerous activities have frontal white matter tracts that are more adult in form than their more conservative peers.

## Introduction

Each year, according to the CDC, 27,000 people between the ages of 10 and 24 die from bad decisions – primarily accidents, homicide, and suicide [1]. The adolescent years, in particular, are a period of heightened vulnerability to reckless behavior that occurs despite the fact that adolescents are more cognitively mature than children [2]. Actuarial tables indicate that adolescents and young adults are more likely to drive recklessly and are more likely to drive under the influence of alcohol or drugs [3]. Viewed in hindsight, many of these adverse outcomes seem to be a result of a poor decision. Although nobody is immune from making bad decisions, adolescents and young adults seem to make a disproportionate share of ultimately fatal or debilitating ones; indeed, bad decisions are the greatest cause of morbidity and mortality in adolescents.

There are two broadly different theories about why adolescents might engage in risky, or dangerous, behaviors. Both theories are informed by the biological changes that occur during adolescence, including both sexual development and neurobiological changes in the brain. One theory suggests that risk-taking in adolescence results from a mismatch in maturity between emotional systems and cognitive control systems of the brain [4]–[6]. According to this dual-systems theory, development of hedonic drive systems (e.g. sensation-seeking) occurs early in adolescence which is then followed later in development by cognitive control systems [7], [8]. Recent evidence suggests a complex temporal development pattern of both emotional and cognitive skills, but linking these development patterns to the real-world decisions that adolescents make has been challenging. Most adolescents navigate the teen years without major problems or injuries, but for those that do not, the period of mid-adolescence (age 15–19) is the time when they are more likely to engage in high-risk behaviors [9], [10]. But whether this is due to an increase in sensation-seeking or lack of development in impulse control is not resolved. Cross-sectional studies of cognitive and emotional function suggest that sensation-seeking follows a curvilinear trajectory, also peaking in mid-adolescence, while impulse control develops linearly into adulthood [7]. However, even with large cohort studies, individual variation remains the rule, and understanding why an individual adolescent engages in dangerous behaviors is complex and not solely a function of chronological age.

Cross-sectional and longitudinal MRI studies have revealed a dynamically changing pattern of gray-matter density from childhood to early adulthood, which are broadly consistent with the dual-systems theory. The prefrontal regions, being the last to show evidence of anatomical change, may not be fully “mature” until the mid-20's [11]–[13]. Less is known about the development of subcortical reward systems, although fMRI studies have suggested that nucleus accumbens responses tend to be higher in adolescents than both children and adults, which may explain adolescents' likelihood of engaging in risky behaviors [14]–[16]. In addition to the dual-systems theory, an alternative theory posits that puberty leads individuals to biological maturity sooner than society permits, resulting in a “maturity gap” [17], [18]. Engagement in high-risk behaviors, according to this theory, is an attempt by the adolescent to bridge this gap by achieving autonomy [19]. An important distinction between these two theories lay in whether it is the immature or mature brain that is associated with adolescent risk-taking. Cross-cultural studies of adolescent behavior, for example, do not find a universal surfeit of risk-taking during the adolescent period, calling into question whether adolescent risk-taking has its origins in biology [18], [20].

To date, the brain imaging research that has focused on the adolescent brain and risk-taking has been of either gray matter changes or functional (fMRI) differences. A relatively new entrant into the imaging toolbox is the use of diffusion tensor imaging (DTI) to measure structural changes in white matter, which is another important determinant of brain maturity. Developmental changes of gray matter and white matter follow different trajectories, and the relationships between gray and white matter and fMRI activation are not known [21]. In general, maturation of white matter in children and adolescents is associated with an increase in both myelination and fiber density [22]. Recent applications of DTI have confirmed this finding, demonstrating that DTI is a valid tool for charting developmental changes in white matter [23]–[27]. Interestingly, the precise localization of white matter tracts undergoing age-related changes varies markedly across these studies. We hypothesize that an important source of this variance is the degree of intellectual, emotional and social maturity of the adolescent himself. Adolescents who engage in activities that are more mature (or dangerous) for their chronological age may have structurally different brains than their relatively immature (or conservative) peers. The question is: what is the true relation between dangerous behavior and white matter maturity? To answer this question, we correlated DTI measures of white matter organization with an age-adjusted measure of real-world engagement in dangerous behaviors in a cohort of adolescents (N = 91, age 12–18). We find evidence suggesting that engaging in dangerous behaviors in adolescence is associated with greater (not less) frontal white matter maturity.

## Results

To measure engagement in dangerous behaviors, we used the Adolescent Risk Questionnaire (ARQ), which is a 22-item survey of activities such as drinking and driving, driving without a license, having unprotected sex, and taking drugs [28]. Previously, the ARQ was normed in a sample of 970 adolescents [28], and principal component analysis identified four factors: 1) thrill-seeking behaviors (e.g. snow skiing, inline skating, parachuting); 2) rebellious behaviors (e.g. drinking, smoking, taking drugs); 3) reckless behaviors (e.g. drinking & driving, having unprotected sex, speeding); and 4) antisocial behaviors (e.g. cheating, teasing people). Because not all of the items on the ARQ are dangerous, we used a subset of 10 that captured contemporary dangerous behaviors in teens. These are the items identified by Gullone et al. as factors 2 and 3 (rebellious and reckless behaviors) minus “stealing cars” but with the addition of “sniffing gas or glue” (see Methods for further details). The score on each item ranged from 0 (never done) to 4 (done very often), and the total score was expressed as a fraction out of a possible 40. As a crude check of the validity of the ARQ, we compared the responses on item 20 (taking drugs) to the urine drug screens. Nine subjects tested positive for marijuana, and all but one of them indicated on the ARQ that they had taken drugs. The median response of this group was 3 (“often”), compared with the median response of 0 in the rest of the cohort (Mann-Whitney U test, *P*<0.0001).

**Figure 1:**
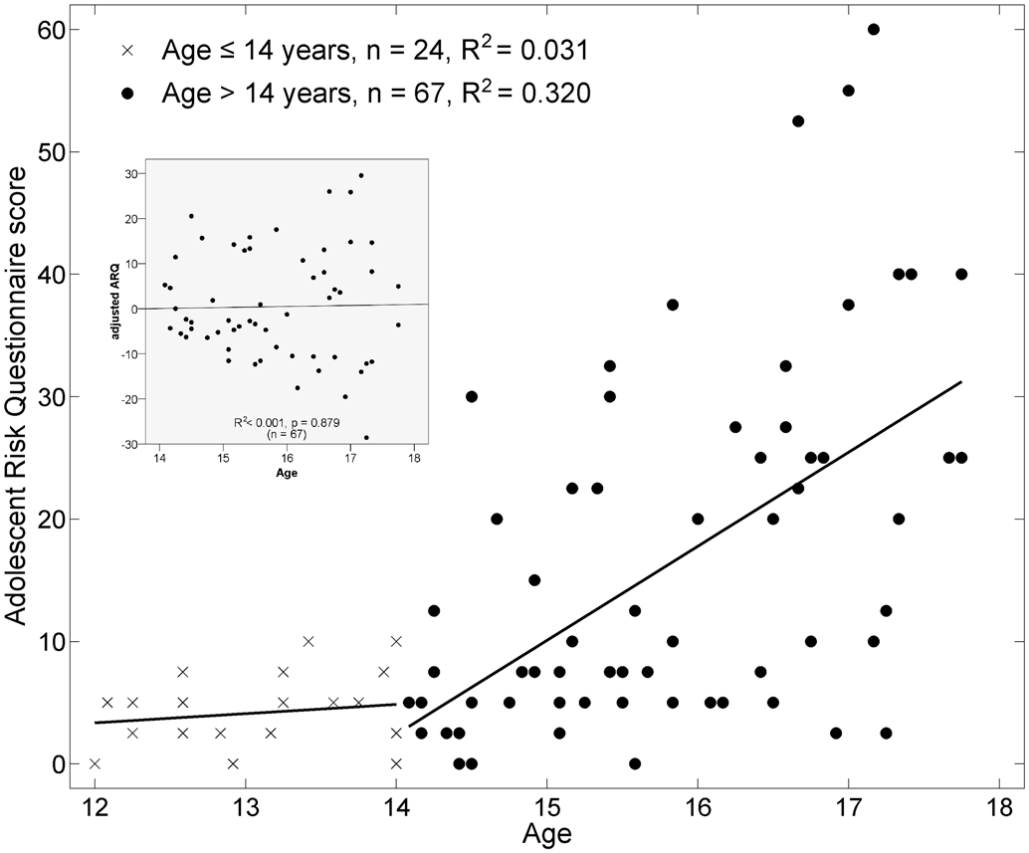
Relationship of Adolescent Risk Questionnaire (ARQ) score to chronological age. The ARQ score ranges from 0–100 and is expressed as a percentage out of the maximum possible score. Prior to age 14, there was very little variation in the ARQ. Beginning at age 14, there was a linearly increasing trend with age and also an increase in the variance. DTI analyses were subsequently performed on the >14 cohort after adjusting the ARQ for the age trend. The residuals of this linear regression (*AdjARQ*) were checked to make sure that they were not correlated with age (*inset*), which they were not (R^2^<0.001, *P* = 0.879).

Not surprisingly, we observed a significant relationship between chronological age and ARQ (Fig. 1). Prior to approximately age 14, there was very little variation in the ARQ score, which is not surprising given the low level of engagement in these activities in 12 and 13 year-olds. Beginning at about age 14 – the approximate age at which adolescents enter high school – we observed both a positive age-related trend in ARQ as well as an increase in the variance. Thus, adolescents show a greater differentiation in their engagement in dangerous activities beginning at age 14. We hypothesize that the variation in dangerous activity engagement is linked to structural differences in white matter.

Brain imaging was performed on a Siemens 3T Trio. Diffusion-sensitizing gradient encoding was applied in 12 directions with a diffusion-weighting factor of b = 1000 s/mm^2^. One (b0) image was acquired without a diffusion gradient (b = 0 s/mm^2^). Six sets of each image were acquired and subsequently averaged. Processor-intensive portions of the DTI analysis were run in parallel on a 1,024-core Sun Grid Engine computing cluster. Using FDT 2.0 (FSL, www.fmrib.ox.ac.uk/fsl), DTI datasets were first eddy current and motion corrected. Using FSL's FLIRT tool, registration parameters were calculated to and from each subject's T1, average b0 image, and an MNI template, and were saved as affine matrices (six transformations were computed in total). Voxelwise values of fractional anisotropy (FA), axial diffusivity (λ_1_, or AD), and transverse diffusivity ((λ_2_+λ_3_)/2, or TD) were calculated. We used tract-based spatial statistics (TBSS as implemented in FSL) to perform both voxelwise and clusterwise statistical correlations between local diffusion indices and subjectwise measures for age, sex, and adjusted-ARQ score [29]. After aligning individual FA maps to the template, a cross-subject mean FA image was calculated and used to generate a white matter tract skeleton (threshold at FA>0.2). Individual subject FA values were then warped onto this skeleton for statistical comparisons. The same was done for AD and TD.

To test for local correlations between ARQ and diffusion measures, we used threshold-free cluster enhancement (TFCE) [30]. TFCE is implemented in the FSL program and uses the randomize program to perform permutation-based testing [31]. The TFCE statistic is based on an “area-under-the-curve” metric that combines the height of the unthresholded t-image with its spatial extent. The statistics were built up over 10,000 random permutations with the maximum TFCE recorded at each permutation. The 95^th^ percentile of this distribution was then used as a TFCE-threshold and the significance level calculated from this distribution. Thus, the maps were fully corrected for familywise error at *P*<0.05. This part of the analysis required approximately 2 hours on the cluster (2,000 CPU-hours).

**Figure 2:**
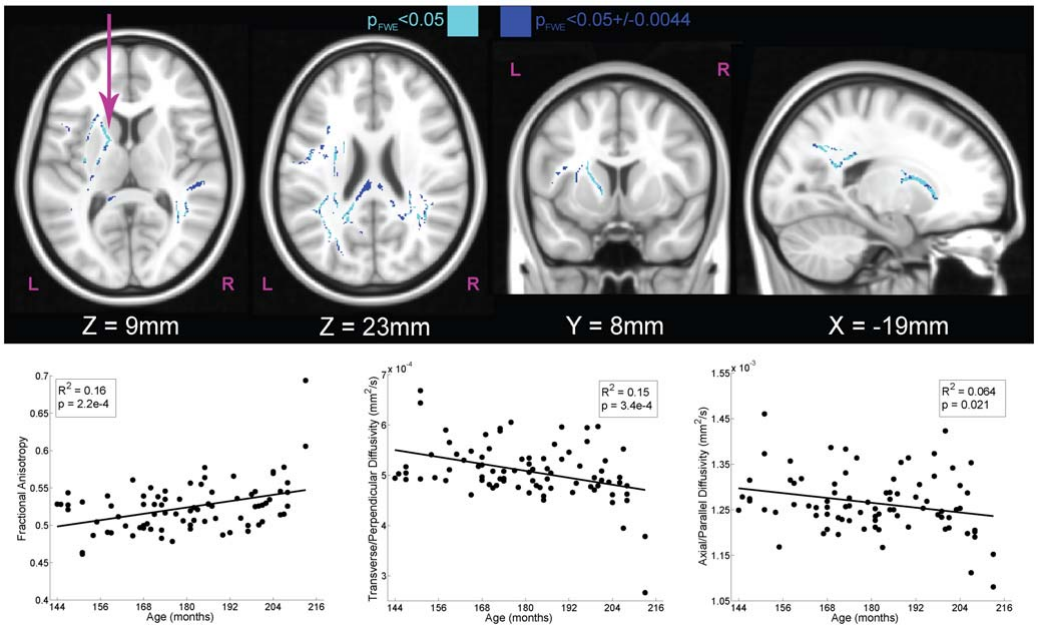
White matter tracts with significant correlations of DTI components to chronological age (N = 83). Using the map for transverse diffusivity (TD), we used tract-based spatial statistics to identify regions within skeletonized white matter tracts that were significantly correlated with age. The maps were fully-corrected for familywise error (FWE) and thresholded at *P*<0.05 (*light blue*). Because significance was determined using a permutation test based on 10,000 samplings of the dataset, the exact level of significance had a margin of error. The 95% confidence interval on this statistic (±0.0044) is also shown (*dark blue*). The scatter plots illustrate the relationship between participant age and component DTI measures for the left anterior internal capsule (*arrow*). Increasing chronological age was associated with increases in fractional anisotropy (FA), which was due primarily to a decrease in TD (*middle bottom*).

First, we identified regions in which there was a significant relationship of chronological age to white matter structure, irrespective of ARQ (Fig. 2). These regions included the left superior fronto-occipital fasciculus and anterior limb of the internal capsule as well as posterior aspects of the corpus callosum (Table 1) [32]. Importantly, the direction of correlation of the DTI components revealed a positive correlation of age with FA (R^2^ = 0.16, *P* = 0.0002, N = 83) and a negative correlation of age with TD (R^2^ = 0.15, *P* = 0.0003, N = 83), which is consistent with previous findings of age-associated changes in white matter [23], [27]. These temporal patterns provided a template for developmental changes in white matter across adolescence.

**Table 1:** Regions correlated with chronological age (months) and Transverse Diffusivity (TD).

TFCE FWE-corrected p	uncorrected p	uncorrected T	X	Y	Z	Size at p_FWE_<0.05 (voxels)	Region
0.0450	0.00010	4.28	−21	6	19	1112	**Left Superior Fronto-Occipital Fasciculus**/Anterior Limb of Internal Capsule
0.0472	0.00284	2.77	−34	−68	27	1858	**Left Superior Parietal**
0.0478	0.00010	4.06	35	−50	19	456	**Right Superior Longitudinal Fasciculus**/Right Posterior Thalamic Radiation (includes Optic Radiation)/Right Posterior Corona Radiata
0.0496	0.00376	2.84	40	−39	17	184	**Right Superior Longitudinal Fasciculus**
0.0494	0.00010	4.31	−50	−2	16	100	**Left Precentral**
0.0498	0.00091	3.34	−37	11	17	40	**Left Superior Longitudinal Fasciculus**
0.0499	0.00112	3.40	−41	11	14	13	**Left Inferior Frontal**

All correlations are negative, and only those clusters surviving TFCE FWE-correction at P<0.05 are listed. Coordinates are in MNI-space (mm).

Second, we performed an independent analysis to determine the brain regions that correlated with ARQ. To account for the known correlations between age and risk-taking we first adjusted the ARQ measure for its relationship to age and sex, and then we correlated this adjusted-ARQ with the DTI components in the participants age 14–18 (the substratum with significant variation in ARQ). Thus, ARQ was fully orthogonalized to other potential explanatory variables like age and sex. As a check of the orthogonalization, the residuals were found to be uncorrelated with age (inset, Fig. 1). We also checked for heteroscedasticity (nonconstant variance with age) and found that higher ages were associated with higher variance in ARQ (Levene's test: *F* = 8.17, *P* = 0.006), but given the large sample size, this should not affect the estimate of the regression line [33]. We confirmed this by repeating the regression with weighted-least-squares and noted that the regression lines were the same.

**Figure 3:**
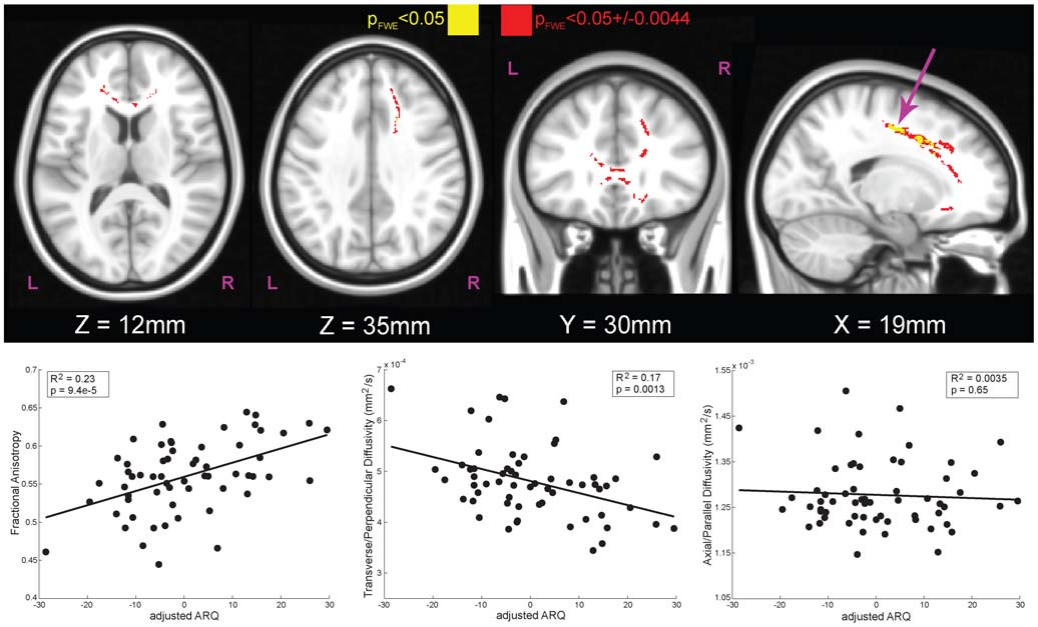
White matter tracts with significant correlations of DTI components to adjusted-ARQ in adolescents 14 years and older (N = 60). The effect of age and sex was first removed from ARQ (Fig. 1), yielding a score that captures engagement in dangerous behaviors independent of sex and chronological age. Using the map for fractional anisotropy (FA), we used tract-based spatial statistics to identify regions within skeletonized white matter tracts that were significantly correlated with adjusted-ARQ. Main effects for sex and age were also included in the DTI model so that the regions identified as being correlated with adjusted-ARQ were purely ARQ-related. The maps were fully-corrected for familywise error (FWE) and thresholded at *P*<0.05 (*yellow*). Because significance was determined using a permutation test based on 10,000 samplings of the dataset, the exact level of significance had a margin of error. The 95% confidence interval on this statistic (±0.0044) is also shown (*red*). The scatter plots illustrate the relationship between adjusted-ARQ and component DTI measures for the right superior corona radiata (*arrow*), but the pattern was the same for all significant regions. Increased ARQ was associated with increased fractional anisotropy (FA, *left bottom*), which was due primarily to a decrease in TD (*middle bottom*). These results show that adolescents who engage in dangerous activities have more mature frontal white matter tracts relative to their conservative peers.

**Figure 4:**
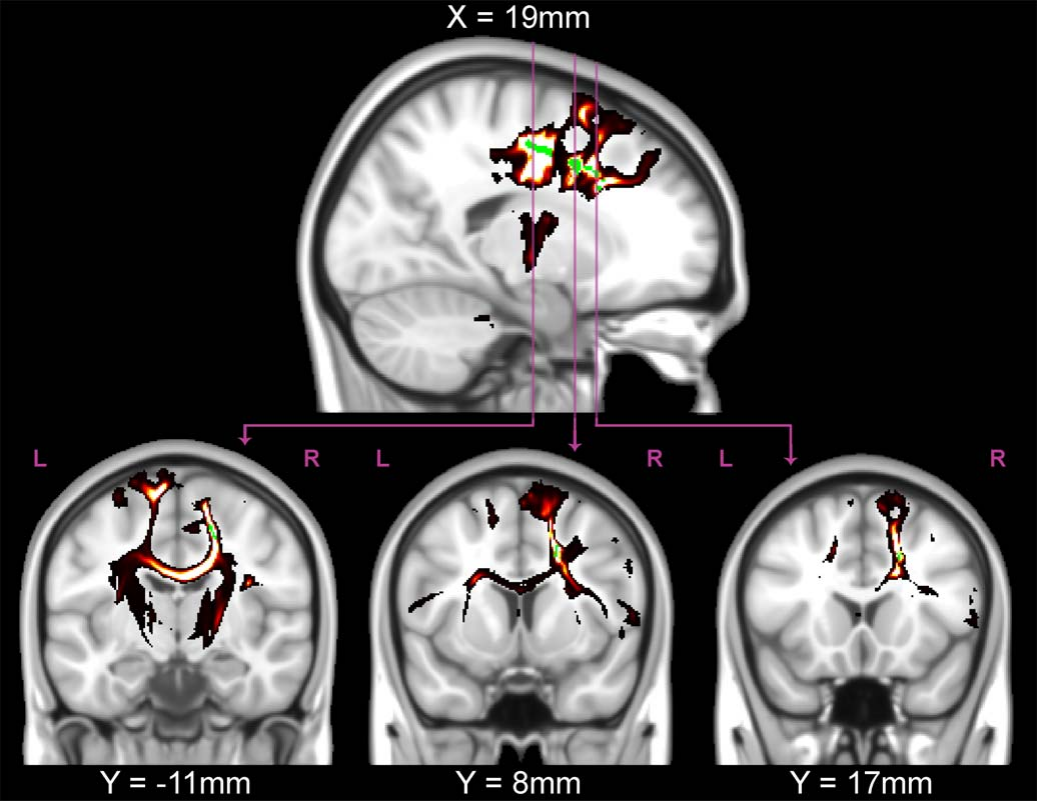
Probabilistic tractography from a typical participant. The significant (*P*<0.05, *green*) TFCE-corrected regions from the FA correlation with adjusted-ARQ were used as seeds for tractography in the participant with the median adjusted-ARQ (female, age 16 years). Tractography revealed that the white matter regions that were significantly correlated with FA were located predominately in descending tracts from prefrontal cortex through the internal capsule, interhemispheric fibers connecting left and right homologous regions through the corpus collosum, and fibers connecting prefrontal regions with the temporal lobe (*lower middle*).

Using a stereotaxic atlas, regions that correlated with adjusted-ARQ included the anterior portion of the corpus callosum and the right superior corona radiata (Fig. 3, Table 2) [32]. Of note, the relationship of adjusted-ARQ was positive with FA (R^2^ = 0.23, *P*<0.0001, N = 60) and negative with TD (R^2^ = 0.17, *P* = 0.0013, N = 60). The location of the white matter tracts demonstrating these relationships points strongly to the tracts that connect the frontal lobes to each other as well as descending tracts (Fig. 4). Furthermore, detailed analysis of the components of FA indicated that the increases in adjusted-ARQ were driven primarily by decreases in TD. Such decreases are suggestive of an increase in density of the fiber bundles and/or increased myelination, both of which are generally associated with increasing chronological age [24], [27]. From this, we infer that adolescents who engage in dangerous behaviors have more mature frontal white matter tracts than their conservative chronological peers.

**Table 2:** Regions correlated with adjusted-ARQ (*AdjARQ*) and Fractional Anisotropy (FA).

TFCE FWE-corrected p	Uncorrected p	Uncorrected T	X	Y	Z	Size at p_FWE_<0.05 (voxels)	Region
0.0490	0.00061	3.30	19	−10	47	116	**Right Superior Corona Radiata**
0.0495	0.00051	3.70	19	8	38	30	**Right Superior Corona Radiata**/Right Anterior Corona Radiata
0.0499	0.00348	2.79	19	18	29	35	**Right Anterior Corona Radiata**

All correlations are positive, and only those clusters surviving TFCE FWE-correction at P<0.05 are listed. Coordinates are in MNI-space (mm).

**Figure 5:**
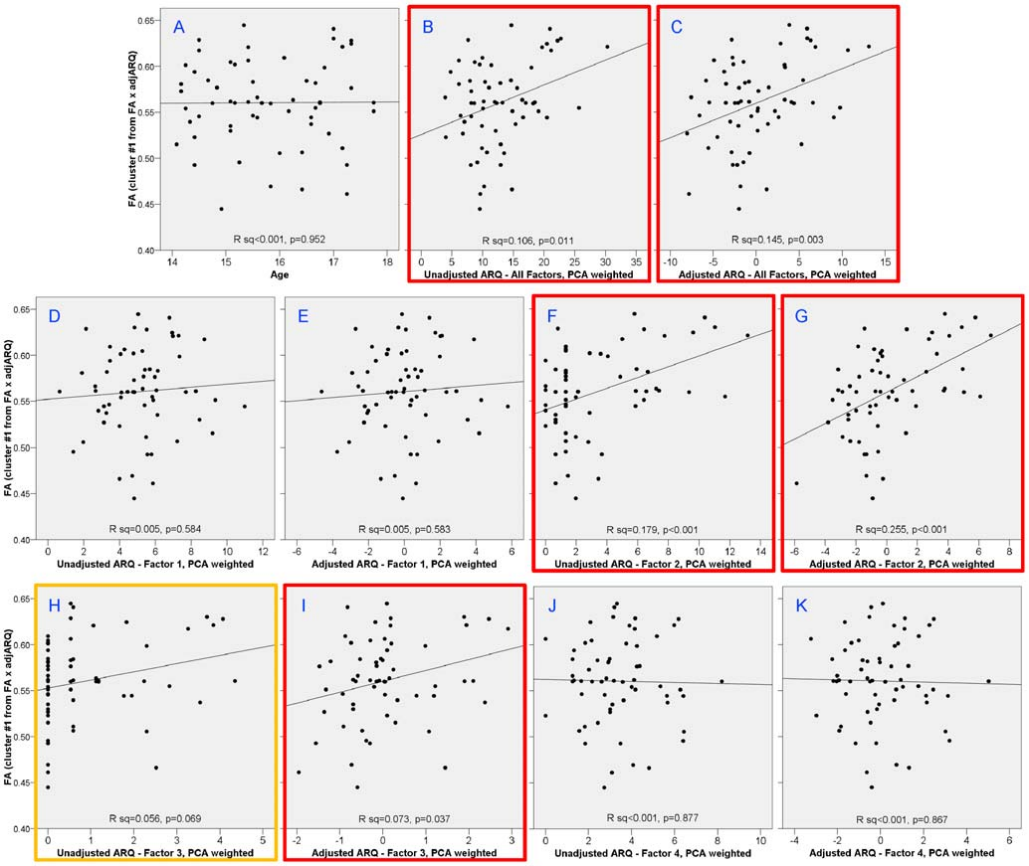
Correlations with FA in right superior corona radiata cluster with age and different factors of the ARQ. Age was uncorrelated with TD in this region (A). Total ARQ was significantly correlated with TD both before (B) and after adjusting ARQ for sex and age (C). Analysis of the subfactors showed that this correlation was driven predominately by factor 2 – rebelliousness (F and G) and slightly to factor 3 – recklessness (H and I).

**Figure 6:**
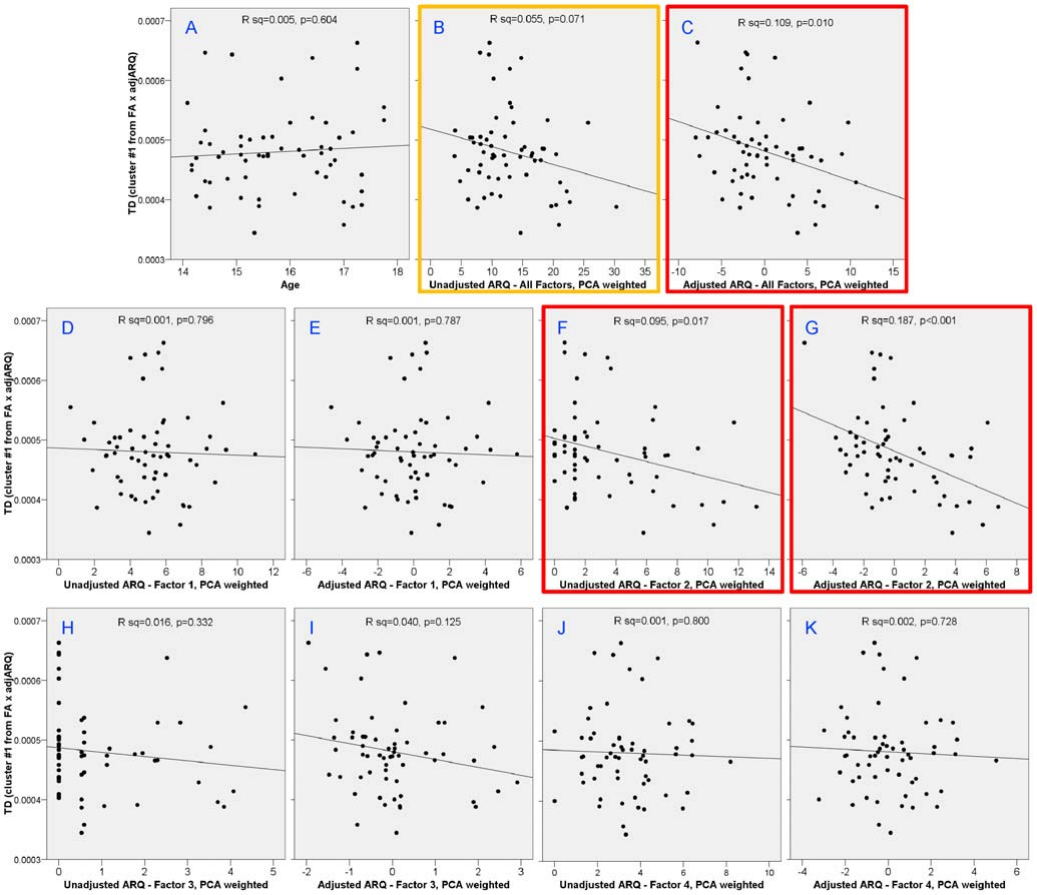
Correlations with TD in right superior corona radiata cluster with age and different factors of the ARQ. Age was uncorrelated with TD in this region (A). Total ARQ was significantly correlated with TD after adjusting ARQ for sex and age (C) and with a trend toward signficance in the unadjusted total ARQ (B). Analysis of the subfactors showed that this correlation was driven predominately by factor 2 – rebelliousness (F and G).

To check that the results were not a spurious correlation from the choice of ARQ items, we performed correlations of FA and TD from the cluster in the right superior corona radiata with the total ARQ score and each of the four PCA factors (Figs. 5 and 6): 1) thrill-seeking; 2) rebelliousness; 3) recklessness; and 4) anti-social behavior. This was done on both raw and sex-and-age-adjusted scores. In addition to our previously defined subset of items, the total ARQ was significantly correlated with both FA (unadjusted: *P* = 0.011; adjusted: *P* = 0.003) and TD (unadjusted: *P* = 0.071; adjusted: *P* = 0.010). Subfactor analysis revealed that these correlations were driven predominately by factor 2, rebelliousness in both FA (unadjusted: *P*<0.001; adjusted: *P*<0.0001) and TD (unadjusted: *P* = 0.017; adjusted: *P*<0.001). Also, to check that the correlations were not confounded with age, we confirmed that there was no significant correlation of age in this region with either FA (Fig. 5A; *P* = 0.952) or TD (Fig. 6A; *P* = 0.604).

Finally, we checked the sensitivity of the results to potential outliers. Although the DTI analysis was permutation-based and therefore robust to outliers, DFBETAS were calculated for the *AdjARQ* x FA regression shown in Fig. 3 (lower left) to identify outlying points that had an excessive amount of influence on the *AdjARQ* regression coefficient. Two points were identified that had suspiciously high standardized DFBETAS of >2/sqrt(N). The regression was performed a second time without these 2 data points (N = 58). The R^2^ for this new regression decreased marginally, from 0.23 to 0.21, with a new significance level of *P* = 0.0003. The regression coefficient for *AdjARQ* also changed only slightly, from 0.00186 to 0.00187. Since these regression plots were primarily for display purposes, removal of the suspected outliers did not result in a significantly different regression coefficient, and the analysis performed to identify the regions displayed was permutation-based, we did not feel that it was necessary to exclude these two participants from the dataset.

## Discussion

These results provide empirical evidence for a neurobiological link to adolescent risk-taking. According to the dual-systems theory of development, adolescent engagement in dangerous activities is believed to result from differential development of hedonic drive processes and impulse control. The latter, in particular, has been thought to result from an immature prefrontal cortex [5], [6], [34]. Our results suggest a partial revision of this theory: adolescents who engage in dangerous behaviors may be more impulsive or more sensation-seeking, but they also have more mature frontal white matter tracts (as evidenced by increased FA and decreased TD) relative to their conservative age-matched peers. Indeed, a growing body of literature consistently favors the interpretation that increased FA, mediated primarily by a decrease in TD, reflects more coherently bundled myelinated axons. This, in turn, is positively associated with cognitive performance [35]–[38].

We observed a strong correlation of ARQ with both FA and TD in the right superior corona radiata and genu of the corpus callosum. The corona radiata includes both ascending (thalamocortical) and descending (corticothalamic and corticofugal) pathways [32], [39]. A similar structure-function relationship of this region has been linked to reading ability and temporal discounting for money [25], [40], [41]. However, the relationship of these fiber bundles to risk-taking is likely to be more general, including both cognitive and motor components as suggested by its anatomical location. The wide swath of the corona radiata that we identified as being linked to dangerous activities connects the cortex with the brainstem and spinal cord, and can therefore be characterized as containing the tracts that connect thought to action. Similarly, we observed significant correlations between adjusted-ARQ and both FA and TD in the genu of the corpus callosum. Tracing the arc of these fibers points to both a superior pathway connecting the left and right prefrontal cortex and an inferior pathway, connecting the left and right orbitofrontal cortex. Presumably the superior pathway relates more to cognitive control function and the inferior pathway to hedonic drives. When we compared adjusted-ARQ to the difference in DTI measures of these two pathways, we did not find a significant correlation either TD or FA of these superior and inferior frontal tracts, indicating that the ARQ-related structural changes were not the result of an imbalance between cognitive control and hedonic brain circuits (at least at the usual level of statistical significance). The functions of these regions, together with the thought-action circuit, point to a consistent differentiation of brain regions involved in planning action and computing expected hedonic value [42]–[44]. The finding that these pathways are more mature in adolescents who engage in dangerous behaviors provides strong support for the theory that behavioral exploration, across a wide range of domains, is associated with differences in white matter connectivity. It is difficult to reconcile this increased maturity with the theory that adolescent risk-taking occurs because of immature cognitive control systems. It is still possible that adolescent risk-taking is driven by a hyperactive reward system, but we found no correlations of subcortical white matter tracts with adjusted-ARQ, so we can neither confirm nor reject that component of the dual-systems theory.

One problem of defining behaviors as “risky” is that it is difficult to objectively define the risk with say, drinking and driving, or having unprotected sex. Adults, after all, engage in some of the same activities that were surveyed on the ARQ (e.g. speeding, having unprotected sex, or getting drunk). Although we tend to think of these activities as less risky for an adult, this creates a conundrum of defining risk (or dangerousness) based not on the objective attributes of the activity but on the person engaging in them. Some have argued that adolescents actually evaluate the risks of these activities the same as adults do, but the reason they engage in them is because they value the outcomes more highly [45]–[47]. Adolescents who engage in these behaviors obtain more experience in a variety of domains that may be plausibly linked to frontal lobe function: social cognition, relationships, and risk-reward trade-offs. Their more conservative peers, in contrast, do not have as much “life experience” and therefore might be expected to have more immature brains. In this interpretation, it is the environmental experience that drives the maturation in the frontal tracts that we observed.

On the other hand, substantial evidence has accumulated that genetic factors account for the majority of differences in brain morphometry in adolescents [48]. These results suggest that biological factors may dominate the rate of maturation of the white matter tracts observed in our study. If true, then precocious development of these tracts may predispose some adolescents to engage in behaviors that society considers too adult in nature for their chronological age. This interpretation is consistent with one theory of adolescent delinquency, in which puberty leads individuals to biological maturity sooner than society permits [17]–[19]. Correlation with the factors of the ARQ provide further insight into the nature of the relationship to white matter. Although significant correlations of both FA and TD were observed with the full-scale ARQ, detailed analysis indicated that this was driven predominately by factor 2 (rebelliousness). The rebellious aspect of adolescent risk-taking may be qualitatively different than the type of risk-taking measured in other studies and may have little to do with impulse control and more to do with sensation seeking, which tends to increase in mid-adolescence [7].

Even so, we observed a large amount of heterogeneity in our sample. Consistent with anthropological studies of adolescence, we found that the majority of our participants were not overtly risk-seeking or delinquent [20]. Although we found a significant relationship of the ARQ to chronological age, only 32% of the variance of the ARQ was actually accounted for by age and sex. The white matter correlations were even less, accounting for a maximum of 25% of the variance in the ARQ subscales, which is typical for a brain imaging study. The degree of subject heterogeneity, therefore, indicates that other unknown factors contribute to a particular individual's propensity to engage in dangerous behaviors. It also underscores the danger in overgeneralizing about the relationship of the adolescent brain to such behaviors.

One limitation of our study is the relatively narrow age range we studied. For the ARQ-related analyses we limited the sample to those participants between 14 and 18. Thus, our study should be viewed as a cross-sectional snapshot of mid-adolescence. Even within this narrow range, significant age effects are known to occur across biological, cognitive, emotional, and social domains. Thus, it is important to take care in accounting for any age effects. In our analyses, age can enter into the results in two ways: 1) as a direct effect on brain development; and 2) through its relationship to behavior as measured on the ARQ. With regard to the direct effects of age on the brain, we observed regions of white matter that showed age-related changes; however, these were different regions from those that showed ARQ-related changes. As a confirmatory check, the ARQ-regions did not show any correlation with age on either FA or TD (R^2^ = 0.005). With regard to the indirect effects of age via the ARQ, we adjusted for age using linear regression. Although other investigators have found evidence for nonlinear effects of age on risk-taking [7], we found no evidence for this over this narrow age range. Although age was correlated with ARQ, the correlation was not to the extent that it would cause collinearity problems (R^2^ = 0.32). A check of the residuals indicated no remaining correlation with age and no need for including nonlinear terms.

Another limitation of our study is that the measure of risk taking, the ARQ, relies on self-report which may be associated with a reporting bias. This is a limitation with most instruments that attempt to assess behaviors that may be perceived as socially unacceptable. However, a similar instrument, the Youth Risk Behavior Surveillance System (YRBSS), has been found to be moderately reliable in adolescents, meaning there is reasonable consistency in how subjects answer the questions on different occasions [49]. Reliability, however, does not necessarily mean that an instrument is valid. There are different types of validity, which include face validity (does the instrument measure what it appears to measure?), criterion validity (do scores correlate with other measures of the behavior being studied?), construct validity (does the instrument make accurate predictions about behavior?), and discriminative validity (does the instrument accurately categorize different groups?) [50]. With regard to the behaviors surveyed on the ARQ, there is little data on validity, and, in general will be difficult to assess in any setting. Self-reports of drug and alcohol use, for example, can be verified through drug screens, but only within a narrow window of time. Even so, we performed urine drug screens on all subjects, which in some settings may increase the likelihood of truthful reporting and which we found to be highly correlated with the urine drug screen we performed [51]. In addition, our consent form explicitly stated that all information is confidential and would not be shared with parents. Most importantly, any bias in self-report is likely to be in the direction of under reporting [52], which would tend to affect the high-ARQ subjects more than the low-ARQ subjects. Thus, such a bias works against the experimenter's ability to detect an effect. The fact that we still found relationships of ARQ to white matter structure, even in the face of possible under reporting, only strengthens our findings.

White matter relationships to variables of individual differences is a growing technique in the neurosciences. It is not yet clear what the relationship of white matter changes is to gray matter changes or subject-specific differences in fMRI activation. There is now substantial evidence that all of these measures change in some age-dependent manner as individuals move through adolescence. However, it is not yet clear how these different measurements of brain structure and function relate to cognitive or emotional function, impulse control, or social maturity of adolescents and young adults. Given the relative novelty of DTI measurements, it is possible the white matter matures in a different manner than gray matter. Thus, more work is clearly necessary to sort out these relationships.

Because of the cross-sectional design cannot directly address the important issue of causality, but our results are consistent with the maturity gap theory. Adolescent engagement in dangerous activities is associated with more mature frontal white matter tracts, but it could be the case that precocious brain development leads to precocious behavior, or participation in these activities accelerates the maturation of the brain. Either way, our results suggest that, like adults, there is a broad spectrum of variation in the adolescent brain, and so aggregate conclusions about brain maturity may lead to imprecise generalizations of what is appropriate or inappropriate for adolescents to do [53]. Many questions remain unanswered, the most intriguing of which may be the issue of causality. Future, longitudinal, studies, could measure the accuracy of prospective imaging modalities in predicting engagement in *future* dangerous activities, as well as resolve the respective roles of biology, development of cognitive control, and life-experience.

## Materials and Methods

### Participants

Prior to imaging, participants were screened for the presence of medical and psychiatric diagnoses. All participants were without a previously diagnosed mental disorder, and none were taking medications. A total of 91 participants were studied. In addition to the DTI study, each adolescent participated in one of three fMRI studies, which will be reported elsewhere. The DTI protocol described below was the same regardless of the fMRI task. Eight participants had problems with their DTI images that precluded their use (N = 2: complete loss of data due to scanner malfunction; N = 2: loss of inferior portion of the brain due to movement or incorrect placement; N = 3: loss of superior portion of the brain due to movement or incorrect placement; N = 1: movement-induced stripe artifacts). The remaining participants (N = 83; 47 female, 36 male) ranged in age from 12.0–17.9 years old (mean 15.1). 37 were Caucasian, 39 were African-American, 1 was biracial, and 6 were “Other.” Each participant and his/her parent or guardian gave written informed consent for a protocol approved by the Emory University IRB.

### Procedures

Participants completed the following procedures.

### Childhood Depression Inventory (CDI)

This inventory was added partway through the experiment to screen out participants who might be depressed, (68 of the 83 subjects completed it). No subject met the exclusion criterion of a T-score greater than 70 (clinically depressed).

### Urine test

Screened for illicit substances and pregnancy in females.

### Adolescent Risk Questionnaire (ARQ)

This was the primary measure of engagement in dangerous behaviors (see Supporting Information Document S1). The ARQ is a 22-item survey of activities such as drinking and driving, driving without a license, having unprotected sex, and taking drugs [28]. Participants completed the questionnaire with full confidentiality, and, as part of the consent process, both the participant and his parent were informed that the results would not be shared with the parent. Subjects completed this questionnaire 4 times: 1) in reference to their own behavior; 2) in reference to how risky they thought the behaviors were; 3) in reference to their peers' behaviors; and 4) in reference to how risky their peers thought the behaviors were. We used only the self-behavior ARQ measure for correlation with DTI.

We chose the ARQ because it was one of the only scales that measures real-world risk taking in adolescents and that has been normed in a large cohort of 970 adolescents [28]. Previously, principal component analysis identified four factors: 1) thrill-seeking behaviors (e.g. snow skiing, inline skating, parachuting); 2) rebellious behaviors (e.g. drinking, smoking, taking drugs); 3) reckless behaviors (e.g. drinking & driving, having unprotected sex, speeding); and 4) antisocial behaviors (e.g. cheating, teasing people) [28]. Many of these questions having nothing to do with risk-taking (“overeating” or “flying in a plane”), some are dated (“rollerblading”), and some are dependent on parental income (“snow skiing” – no skiing in Georgia). Consequently, we used a subset of 10 that captured contemporary risky behaviors in teens (questions 1, 3, 5, 8, 9, 10, 14, 15, 16, 20). The subset was defined at the beginning of the study, before the imaging results were computed and were the same items identified by Gullone et al. as factors 2 and 3 (rebellious and reckless behaviors) minus “stealing cars and joyriding” (which we excluded because the conjunction of 2 activities makes it difficult to answer the question, i.e. one could truthfully answer “no” unless both activities were present) but with the addition of “sniffing gas or glue” (which is obviously dangerous) and according to the Gullone analysis could belong in either factor 3 or 4. The score on each item ranged from 0 (never done) to 4 (done very often), and the total score was expressed as a fraction out of a possible 40. To check that the results were not an artifact of the particular subscale chosen, we also performed correlation analyses on the full scale ARQ and each of the PCA factors separately (with and without adjustment for sex and age).

### Scanning

Scanning was performed on a Siemens 3T Trio. Each subject received a T1-weighted structural image (TR = 2600 ms, TE = 3.93 ms, flip angle = 8, 224×256 matrix, 176 sagittal slices, 1 mm cubic voxel size), a DTI scan (TR = 6500 ms, TE = 90 ms, flip angle = 90, FOV = 220 mm, 128×128 matrix, 34 axial slices, 1.7×1.7×2.5 mm voxel size), and whatever functional scans were performed as part of the fMRI portion of the experiment. For the DTI, a diffusion-sensitizing gradient encoding was applied in 12 directions with a diffusion-weighting factor of b = 1000 s/mm^2^, and one (b0) image was acquired without a diffusion gradient (b = 0 s/mm^2^). Six sets of each image were acquired and subsequently averaged. The total scan time was approximately 1 hour for all scans.

### DTI Analysis

Processor-intensive portions of the DTI analysis were run in parallel on a 1,024-core Sun Grid Engine computing cluster. Using FDT 2.0 (FSL, www.fmrib.ox.ac.uk/fsl), DTI datasets were first eddy current and motion corrected. Using FSL's FLIRT tool, registration parameters were calculated to and from each subject's T1, average b0 image, and an MNI template, and were saved as affine matrices (six transformations were computed in total). Voxelwise values of fractional anisotropy (FA), axial diffusivity (λ_1_), and transverse diffusivity ((λ_2_+λ_3_)/2) were calculated.

We used tract-based spatial statistics (TBSS as implemented in FSL) to perform both voxelwise and clusterwise statistical correlations between local diffusion indices and subjectwise measures for age, sex, and adjusted-ARQ score [29]. After aligning individual FA maps to the fmriB58_FA template in MNI space [54], a cross-subject mean FA image was calculated and used to generate a white matter tract skeleton (thresholded at FA>0.2). Individual subject FA values were then warped onto this skeleton for statistical comparisons. The same was done for AD and TD. Since the template image was constructed from adults, we checked to see if there was a potential confound of aligning adolescent brain images to that template. We found that there was no significant correlation of adolescent age with the warping distance to the template (R^2^ = 0.014, P = 0.284).

Two independent DTI models were implemented. The first simply correlated FA, TD, and AD at each voxel of the skeletonized tracts with chronological age in months. The second model aimed to identify correlations with ARQ after adjusting for age and sex effects. This was applied only in the particpants age 14 and older (leaving N = 60) for 3 reasons: 1) 12 and 13 year-olds are in a different social context than older subjects (e.g. middle school vs. high school) and would be expected to be categorically different; 2) 12 and 13 year-olds are less likely to be sexually mature (e.g. the median age of menarche is 12.4) [55]; 3) 12 and 13 year olds did not have significant variation in ARQ, raising questions about its appropriateness for measuring risk taking in this age group.

To account for the potential effects of age, ARQ was adjusted for sex and age using linear regression:
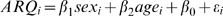
where β's were the regressed coefficients across the cohort, *i* was subject, *sex* was a binary variable (female = 0, male = 1) and *age* was in months. The residuals of this regression, *ε_i_*, represented the adjusted-ARQ for each subject and were fully orthogonalized to sex and age. The sufficiency of the orthogonalization was checked to confirm that the residuals were uncorrelated with age.

The adjusted-ARQ was then used in the following model to determine correlations with the DTI components at each voxel in the skeletonized tracts (e.g. FA):
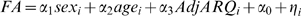
where the α's were the regressed coefficients across the cohort (α_0_ represented the cohort mean), *i* was subject, *sex* was a binary variable (female = 0, male = 1), *age* was in months and *AdjARQ_i_* was that obtained above (*ε_i_* in the behavioral model). Including age as a main effect in the DTI model controlled for age-related changes in white matter independent of ARQ, while using *AdjARQ* as the covariate of interest removed age as a confound on the behavioral parameter. The significance of the coefficient,α_3_, was the primary variable of interest. The same was done for FA, TD, and AD maps.

To test for the significance of local correlations between *AdjARQ* and diffusion measures, we used threshold-free cluster enhancement (TFCE) [30]. TFCE is implemented in the FSL program uses the randomize program to perform permutation-based testing [31]. The TFCE statistic is based on an “area-under-the-curve” metric that combines the height of the unthresholded t-image with its spatial extent. Because relatively little is known about the respective importance of height and extent in DTI data, we chose to weight both equally in the computation of the TFCE statistic (E = 1.0 and H = 1.0). We used 26-neighbor connectivity. The statistics were built up over 10,000 random permutations with the maximum TFCE recorded at each permutation. The 95^th^ percentile of this distribution was then used as a TFCE-threshold and the significance level calculated from this distribution. Thus, the maps were fully corrected for familywise error at *P*<0.05. Subjectwise values of FA, TD, and AD from identified clusters were plotted against *AdjARQ*.

In order to determine the direction of fibers passing through these clusters, those clusters showing significant correlations with *AdjARQ* were also used as seed masks for probabilistic tractography [27], [56]. Using FSL, the probabilistic density function was estimated on the principal fiber direction. Tractography was performed by 5000 streamline samples from each seed voxel. The resultant pathways were volumes at each voxel representing the number of samples passing through that voxel, which, in turn represented the probability of connection to the seed voxel. Spurious connections were removed by setting a threshold of at least 20 samples. Tractography was performed separately for each subject.

## Supporting Information

Document S1 Adolescent Risk Questionnaire
(0.05 MB DOC)
