# Supplementary material for: Adolescent Engagement in Dangerous Behaviors Is Associated with Increased White Matter Maturity of Frontal Cortex
Source: PLoS One. 2009 Aug 26;4(8):e6773. doi: 10.1371/journal.pone.0006773 (PMC2728774; doi:10.1371/journal.pone.0006773)
Supplement: Supplementary file 1 [file pone.0006773.s001.doc]

RISK BEHAVIOUR QUESTIONNAIRE

NB: Items used in our calculation of contemporary ARQ score are boxed.

DIRECTIONS

Below is written a list of behaviours which some people engage in. Read each one carefully circle the word that best describes **your behaviour.**

There are no right or wrong answers.

Remember, circle the word that best describes **your** behaviour about each question in the list.

1. Smoking (tobacco).................................	Never Done	Hardly Ever Done	Done Sometimes	Done Often	Done Very Often
2. Roller blading	Never Done	Hardly Ever Done	Done Sometimes	Done Often	Done Very Often
3. Drinking and driving	Never Done	Hardly Ever Done	Done Sometimes	Done Often	Done Very Often
4. Parachuting	Never Done	Hardly Ever Done	Done Sometimes	Done Often	Done Very Often
5. Speeding	Never Done	Hardly Ever Done	Done Sometimes	Done Often	Done Very Often
6. Stealing cars and going for joy rides........	Never Done	Hardly Ever Done	Done Sometimes	Done Often	Done Very Often
7. Tao Kwon Do fighting............................	Never Done	Hardly Ever Done	Done Sometimes	Done Often	Done Very Often
8. Underage drinking	Never Done	Hardly Ever Done	Done Sometimes	Done Often	Done Very Often
9. Staying out late......................................	Never Done	Hardly Ever Done	Done Sometimes	Done Often	Done Very Often
10. Driving without a licence	Never Done	Hardly Ever Done	Done Sometimes	Done Often	Done Very Often
11. Talking to strangers..............................	Never Done	Hardly Ever Done	Done Sometimes	Done Often	Done Very Often
12. Flying in a plane....................................	Never Done	Hardly Ever Done	Done Sometimes	Done Often	Done Very Often
13. Cheating...	Never Done	Hardly Ever Done	Done Sometimes	Done Often	Done Very Often
14. Getting drunk	Never Done	Hardly Ever Done	Done Sometimes	Done Often	Done Very Often
15. Sniffing gas or glue	Never Done	Hardly Ever Done	Done Sometimes	Done Often	Done Very Often
16. Having unprotected sex	Never Done	Hardly Ever Done	Done Sometimes	Done Often	Done Very Often
17. Leaving school	Never Done	Hardly Ever Done	Done Sometimes	Done Often	Done Very Often
18. Teasing and picking on people	Never Done	Hardly Ever Done	Done Sometimes	Done Often	Done Very Often
19. Snow skiing..	Never Done	Hardly Ever Done	Done Sometimes	Done Often	Done Very Often
20. Taking drugs (including marijuana).......	Never Done	Hardly Ever Done	Done Sometimes	Done Often	Done Very Often
21. Overeating...	Never Done	Hardly Ever Done	Done Sometimes	Done Often	Done Very Often
22. Entering a competition...........................	Never Done	Hardly Ever Done	Done Sometimes	Done Often	Done Very Often
